# Supplementary material for: DisA Restrains the Processing and Cleavage of Reversed Replication Forks by the RuvAB-RecU Resolvasome
Source: Int J Mol Sci. 2021 Oct 20;22(21):11323. doi: 10.3390/ijms222111323 (PMC8583203; doi:10.3390/ijms222111323)
Supplement: Supplementary file 1 [file ijms-22-11323-s001.zip › ijms-1382926-supplementary.pdf]

Supplementary information

DisA restrains the processing and cleavage of reversed replication forks by the RuvAB-RecU resolvasome

Carolina Gándara[1,†,§], Rubén Torres[1,†], Begoña Carrasco[1], Silvia Ayora[1,§] and Juan C. Alonso[1,§]

[1]Department of Microbial Biotechnology, Centro Nacional de Biotecnología, CNB-CSIC, 3 Darwin St, 28049 Madrid, Spain

DisA binds DNA with low affinity at low Mg^{2+} concentration

DisA preferentially binds DNA at physiological Mg^{2+} concentrations (Figure S1A). Since RuvA preferentially binds HJ DNA in the presence of EDTA, and binding is strongly reduced the presence of 10 mM $MgCl_2$ [1-3], and RecU binds HJ DNA in the presence of 1 mM $MgCl_2$ and cleaves the HJ DNA to yield two nicked duplex products in the presence of 10 mM $MgCl_2$ [4,5] the binding affinity of DisA to the different DNA substrate was re-evaluated by reducing Mg^{2+} concentrations.

In the absence of Mg^{2+}, DisA poorly bound HJ-J3 DNA (Figure S1B), and in the presence of 1 mM $MgCl_2$, DisA binds HJ-J3 DNA with ~6-fold lower affinity (p <0.01, K_{Dapp} of 18 ± 1.5 nM) when compared to physiological Mg^{2+} concentrations (10 mM) (Figure S1A-B). When HJ-J3 DNA was replaced by flayed DNA, dsDNA or ssDNA, similar K_{Dapp} values were calculated (Figure S1A-B). Except with dsDNA, large molecular mass complexes that mainly remained trapped in the well were observed. These results suggested that DisA prefers to bind branched DNA, and that the engagement of DisA with dsDNA is different to that with branched DNA, in accordance with DisA scanning the dsDNA and pausing upon a replicative stress *in vivo*.

References

1. Parsons, C.A.; Tsaneva, I.; Lloyd, R.G.; West, S.C. Interaction of *Escherichia coli* RuvA and RuvB proteins with synthetic Holliday junctions. *Proc Natl Acad Sci U S A* **1992**, *89*, 5452-5456, doi:10.1073/pnas.89.12.5452.
2. Ingleston, S.M.; Dickman, M.J.; Grasby, J.A.; Hornby, D.P.; Sharples, G.J.; Lloyd, R.G. Holliday junction binding and processing by the RuvA protein of *Mycoplasma pneumoniae*. *Eur J Biochem* **2002**, *269*, 1525-1533, doi:10.1046/j.1432-1033.2002.02805.x.
3. Panyutin, I.G.; Biswas, I.; Hsieh, P. A pivotal role for the structure of the Holliday junction in DNA branch migration. *EMBO J* **1995**, *14*, 1819-1826.
4. Ayora, S.; Carrasco, B.; Doncel, E.; Lurz, R.; Alonso, J.C. *Bacillus subtilis* RecU protein cleaves Holliday junctions and anneals single-stranded DNA. *Proc Natl Acad Sci U S A* **2004**, *101*, 452-457.
5. Suzuki, Y.; Endo, M.; Cañas, C.; Ayora, S.; Alonso, J.C.; Sugiyama, H.; Takeyasu, K. Direct analysis of Holliday junction resolving enzyme in a DNA origami nanostructure. *Nucleic Acids Res* **2014**, *42*, 7421-7428, doi:10.1093/nar/gku320.

Supplementary Figures S1 to S3

A

Substrate code	Substrate description	Structure + oligonucleotide composition	K_{Dapp} (nM) 10 mM $MgCl_2$	K_{Dapp} (nM) 1 mM $MgCl_2$
HJ	fixed Holliday Junction J3	J3-1 J3-2 J3-4 J3-3	≈ 3	≈ 18
Flayed	60-nt flayed DNA	170 173	≈ 2	≈ 16
80-dsDNA	80-bp dsDNA	J3-2 J3-5	≈ 3	≈ 24
80-ssDNA	80-nt ssDNA	J3-2	≈ 2	≈ 30

B

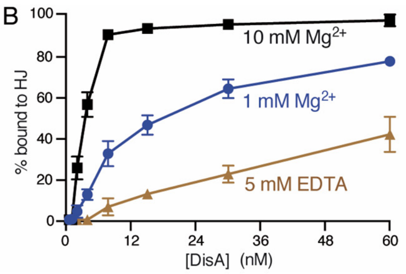

Figure S1. Affinity of DisA for different DNA substrates. (A) The depicted DNA substrates were radiolabelled and incubated with increasing DisA concentrations (by doubling from 0.45 to 60 nM) for 15 min at 37 ºC in buffer C containing 1 or 10 mM $MgCl_2$. 0.2% glutaraldehyde was then added and protein-DNA complexes were separated by EMSA. (B) [γ-^{32}P] HJ DNA was incubated with increasing concentrations of DisA (doubling from 0.45 to 60 nM) for 15 min in buffer C containing 1 mM or 10 mM $MgCl_2$ or 5 mM EDTA at 37 ºC. The DisA-HJ DNA complexes were detected by EMSA, and the fraction of DisA-HJ DNA quantified as described in Materials and methods. The results are shown as the mean ± SEM of the K_{Dapp} value in nM of at least 3 independent experiments.

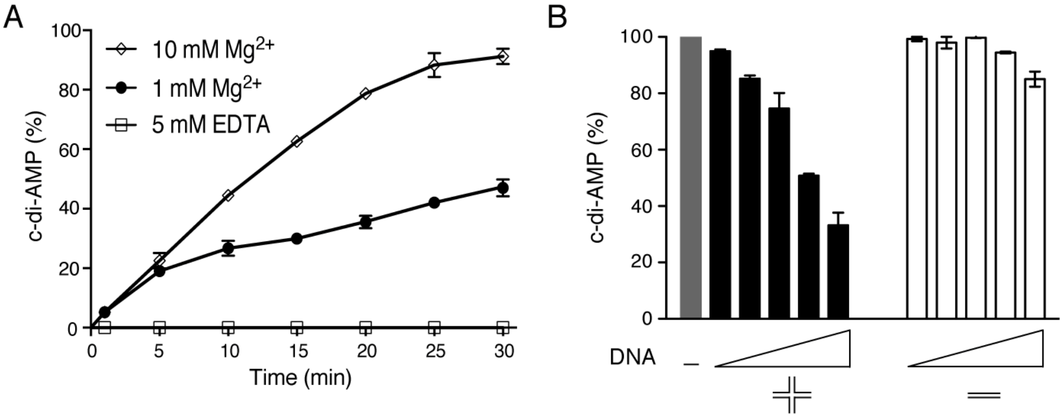

Figure S2. DisA DAC activity requirements. (A) DAC activity is higher at 10 mM Mg^{2+} ions. DisA (60 nM) was incubated in buffer D containing 1 mM $MgCl_2$, 10 mM $MgCl_2$ or 5 mM EDTA and 100 µM ATP (at a ratio of 1:2000 [α^{32}P]-ATP:ATP) at 37ºC. Samples were taken at different times and separated by TLC. Spots corresponding to the ATP and c-di-AMP molecules were quantified and the mean ± SEM of >3 independent experiments is shown. (B) DisA DAC activity is inhibited specifically by binding to HJs. DisA (60 nM) was incubated in buffer D containing 10 mM $MgCl_2$ and 100 µM ATP (at a ratio of 1:2000 [α^{32}P]-ATP:ATP) in the absence or presence of increasing concentrations (doubling from 15 to 240 nM) of HJ-J3 or dsDNA at 37°C for 30 min. Samples were separated by TLC. Spots corresponding to the ATP and c-di-AMP molecules were quantified and the mean ± SEM of >3 independent experiments is shown.

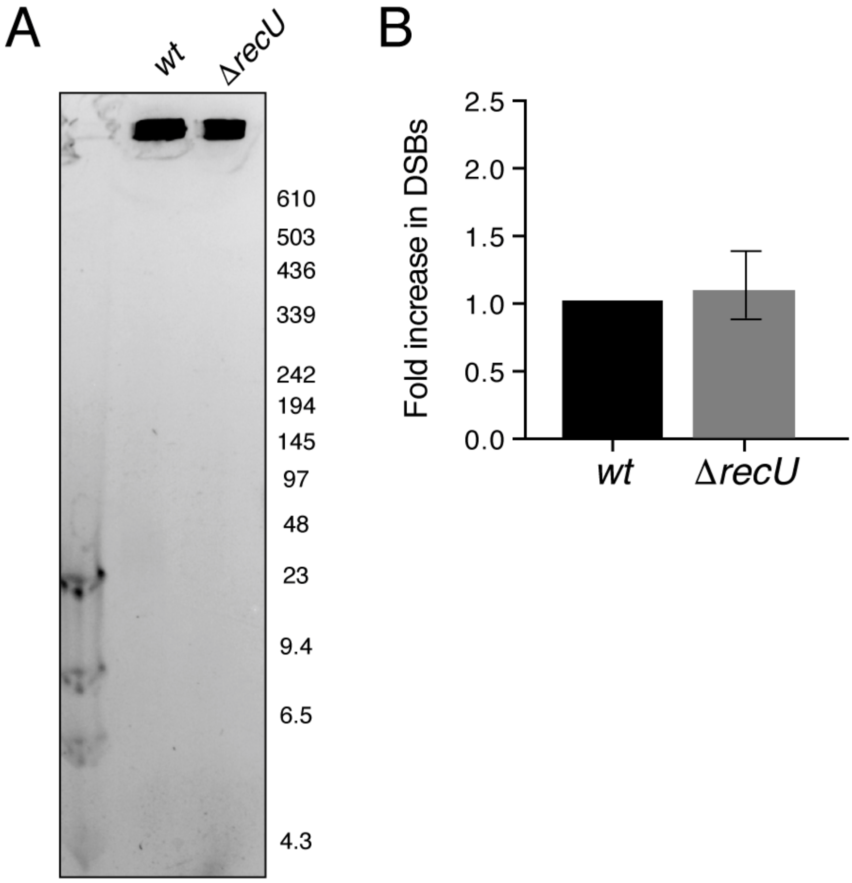

Figure S3. Extent of spontaneous chromosomal fragmentation in the Δ*recU* context. (A) Agarose plugs were prepared from unperturbed *wt* and Δ*recU* cells and were subjected to PFGE. The marker shown is a Lambda DNA *Hin*dIII digest, and the running positions of markers shown on the rigth. (B) Quantification of the chromosomal fragmentation. The amount of chromosomal fragmentation was calculated dividing the signal in the 610- 48 Kb region in a given lane by the combined signal of the lane plus well and results are plotted relative to the signal of the *wt* that has a value of 1. The results shown are the mean of ± SD of three independent experiments.
